# Surprise minimization as a learning strategy in neural networks

**DOI:** 10.1186/1471-2202-16-S1-P77

**Published:** 2015-12-18

**Authors:** Mohammad Javad Faraji, Kerstin Preuschoff, Wulfram Gerstner

**Affiliations:** 1 School of Life Sciences, Brain Mind Institute and School of Computer and Communication Sciences, Ecole Polytechnique Federal de Lausanne (EPFL), CH-1015, Lausanne, Switzerland; 2 Geneva Finance Research Institute, University of Geneva, CH-1211, Geneva, Switzerland

## 

Surprise is informative because it drives attention and modifies learning. Not only has it been described at different stages of neural processing [1], but it is a central concept in higher levels of abstraction such as learning and memory formation [2]. Several methods, including Bayesian and information theoretical approaches, have been used to quantify surprise. In Bayesian surprise, only data observations which substantially affect the observer's beliefs yield surprise [3,4]. In Shannon surprise, however, observations that are rare or less likely to happen are considered surprising [5]. Although each of the existing measures partly incorporates conceptual aspects of surprise, they still suffer from some drawbacks including implausibility from the view point of neural implementation.

We first review the two probability-based surprise measures above, and discuss their pros. We then propose a novel measure for calculating surprise which benefits from the advantages of both measures. Importantly, the proposed measure benefits from calculating surprise during learning phase (e.g., inference about parameters in Bayesian framework). This is in contrast to Bayesian surprise where the surprise calculation is *not *prior to the inference step. Our proposed method can also be neurally implemented in a feed-forward neural network.

Furthermore, we propose a principle of (future) surprise minimization as a learning strategy; that is if something unexpected (surprising) happens, the subjective internal model of the external world should be modified such that the same observation becomes less surprising if it happens again in the not so distant future. We mathematically describe a class of learning rules which obey that principle. We show that standard Bayesian updating and the likelihood maximization technique both belong to such class. It accredits usage of well-known inference techniques in frequentist and Bayesian frameworks from a novel perspective. As a consequence, we propose a modified Bayesian method for updating beliefs about the world. This learning rule also obeys the principle of surprise minimization. In this method, the influence of the likelihood term on the posterior belief can be controlled by a subjective parameter. We apply this technique to learning within changing environments. Modified Bayesian updating helps the learning agent to actively control the influence of new information on learning environments. As a result, the agent quickly adapts to the changing environments.
